# Delayed urticaria during treatment with anti‐CGRP monoclonal antibodies in migraine

**DOI:** 10.1111/head.70082

**Published:** 2026-03-15

**Authors:** Christoph T. Berger, Federico Burguet Villena, Severin B. Vogt, Lukas Heydrich, Karin Hartmann, Athina Papadopoulou

**Affiliations:** ^1^ University Center for Immunology University Hospital Basel Basel Switzerland; ^2^ Translational Immunology, Department of Biomedicine University of Basel Basel Switzerland; ^3^ Department of Clinical Research University Hospital Basel and University of Basel Basel Switzerland; ^4^ Clinic of Neurology University Hospital Basel Basel Switzerland; ^5^ Clinical Pharmacology and Toxicology University Hospital Basel Basel Switzerland; ^6^ Neurology am Kunstmuseum Basel Switzerland; ^7^ CORE Lab, Psychosomatic Competence Center, Department of Neurology, Inselspital Bern University Hospital, University of Bern Bern Switzerland; ^8^ Allergy and Immunity, Department of Biomedicine University Hospital Basel and University of Basel Basel Switzerland; ^9^ Division of Allergy, Department of Dermatology University Hospital Basel and University of Basel Basel Switzerland

**Keywords:** allergy, atopy, CGRP, erenumab, fremanezumab, galcanezumab, migraine, Rimegepant, risk, urticaria

## Abstract

**Objective:**

To characterize clinical presentation and management of urticaria associated with calcitonin gene‐related peptide (CGRP) ‐targeting monoclonal antibodies (mAbs) for migraine prophylaxis.

**Background:**

CGRP‐targeting mAbs are effective in migraine prophylaxis, but have been associated with hypersensitivity reactions, including urticaria. The underlying mechanisms, risk factors, and therapeutic consequences of these anti‐CGRP mAb‐related hypersensitivity reactions remain poorly understood.

**Methods:**

We performed a retrospective case series with descriptive analysis on five patients who developed urticaria after anti‐CGRP mAb administration. Timing of reactions, history of urticaria, re‐exposure strategies including premedication, and clinical outcomes were analyzed by chart review.

**Results:**

Urticaria occurred after the first injection in three patients and after the third or sixth injection in two. Onset was delayed (12–48 h) in all patients, indicating a non‐IgE‐mediated hypersensitivity. Four of five patients had a prior history of urticaria. All patients were re‐exposed: two to the same anti‐CGRP mAb and three to a different. Three patients received H1‐antihistamine premedication. All premedicated patients in this series tolerated re‐exposure, irrespective of switching. One patient experienced worsening urticaria with repeated dosing without premedication despite negative allergy testing, but later tolerated the same anti‐CGRP mAb with premedication. In contrast, urticaria or angioedema recurred in two patients who switched anti‐CGRP mAb without premedication. One subsequently tolerated rimegepant.

**Conclusion:**

Anti‐CGRP mAb‐associated urticaria in this case series was delayed and likely non‐immunoglobulin E (non‐IgE)‐mediated. Our experience supports that in selected patients with delayed urticaria, individualized management, including H1 antihistamine premedication, may allow continuation of effective migraine prophylaxis. Larger cohorts are needed to identify risk factors and to inform general management recommendations.

**Abbreviations**
CGRP: calcitonin gene‐related peptide
COVID‐19: coronavirus disease 2019
FDA: United States Food and Drug Administration
IC: Information Component
IC_25_
: Information Component lower 95% credibility limit
IgE: immunoglobulin E
mAb: monoclonal antibody
PT: preferred term
STROBE: Strengthening the Reporting of Observational Studies in Epidemiology
WHO: World Health Organization

## INTRODUCTION

Migraine is a frequent debilitating neurological disorder that significantly affects quality of life.^1^ The pathophysiology of migraine involves the calcitonin gene‐related peptide (CGRP), a neuropeptide crucial for pain transmission and vasodilation during migraine attacks.^2^ Monoclonal antibodies (mAbs) targeting CGRP or its receptor have emerged as highly effective therapeutic options for episodic^3^, ^4^, ^5^, ^6^ and chronic^7^, ^8^, ^9^ migraine. Currently, approved CGRP‐targeting mAbs include eptinezumab, erenumab, fremanezumab, and galcanezumab. Erenumab is a fully human mAb that blocks the CGRP receptor, whereas eptinezumab, fremanezumab, and galcanezumab specifically target the CGRP ligand, preventing it from binding to its receptor.^2^ All mAbs are administered subcutaneously except for eptinezumab (intravenously).

The safety of anti‐CGRP mAbs has been investigated in controlled clinical trials and post‐marketing surveillance. Mild and self‐limiting injection‐site reactions are common in all anti‐CGRP treatments.^10^ Pharmacovigilance data have documented occurrences of urticaria, angioedema, and other hypersensitivity reactions, albeit at a low frequency.^10^, ^11^ The immunological mechanisms of these reactions and the clinical relevance regarding treatment continuation have, however, not been elucidated.

Thus, we aimed to describe a series of patients who developed urticaria while on anti‐CGRP mAbs and their outcomes. We hypothesized that delayed urticaria following anti‐CGRP mAbs is predominantly non‐immunoglobulin E (non‐IgE)‐mediated and may be mitigated by antihistamine premedication.

## METHODS

This was a single‐center retrospective observational case series. All patients provided written informed consent for the use of their clinical data for publication. The ethics committee of North‐Western Switzerland approved the study (EKNZ project‐ID: 2024–01066), which was conducted in accordance with the Helsinki Convention. Patients were evaluated at the University Hospital Basel between 03/2022 and 08/2025 by the same experienced allergist and clinical immunologist (C.T.B.). Inclusion criteria were: (i) treatment with an anti‐CGRP mAb, and (ii) development of urticaria temporally associated with treatment. Urticaria was defined based on the clinical presentation with or without angioedema. Dermatology consultation was not routinely required. No formal severity grading scale was applied. A retrospective chart review was performed to extract baseline data on the clinical course of migraine, prior therapies, and a history of urticaria in other settings. Data on clinical reactions were extracted, with particular focus on the timing and duration of urticaria, diagnostic tests, and subsequent re‐exposure strategies, including premedication. Notably, no predefined protocol for the evaluation or management of urticaria was in place. Clinical decisions were made on a case‐by‐case basis. In the first cases, treatment was generally switched due to uncertainty regarding the mechanism; in the later cases, continuation with premedication was favored, reflecting the assumption of a class effect. When premedication was prescribed, we used bilastine (20 mg orally) once daily starting 1 day before and continuing for 3 days after each anti‐CGRP mAb administration, as previously applied to prevent urticaria following mRNA coronavirus disease 2019 (COVID‐19) vaccination.^12^ The primary outcome was the recurrence of urticaria following anti‐CGRP mAb exposure.

We inquired the pharmacovigilance data from the World Health Organization (WHO) database (VigiBase; ATC code N02CD (CGRP antagonists) and preferred terms (PT) “urticaria”) on 17 October 2025 to test for a urticaria safety signal in patients treated with anti‐CGRP mAbs.

All Data were analyzed descriptively using Excel and GraphPad Prism (Version 10.4.2). We used the Strengthening the Reporting of Observational Studies in Epidemiology (STROBE) reporting checklist for preparing the manuscript.^13^

## RESULTS

**FIGURE 1:**
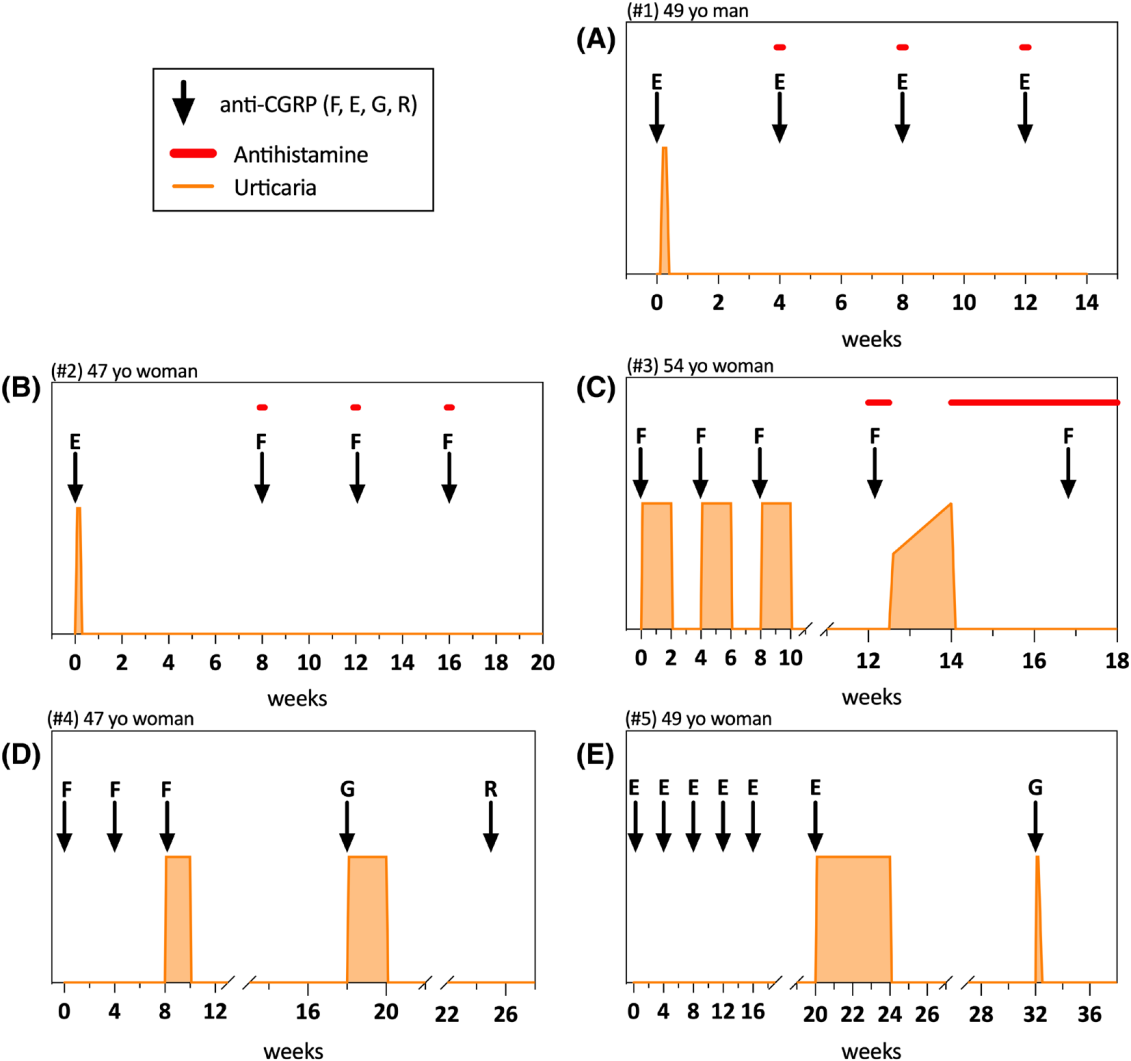
Initial reaction and clinical outcome following re‐exposure to anti‐CGRP mAbs. Arrows indicate dose administration of the indicated anti‐CGPR mAbs: E, erenumab; F, fremanezumab; G, galcanezumab; R, rimegepant (oral CGRP receptor antagonist). The orange line indicates the occurrence and duration of urticaria. Red dots/bars indicate premedication with H1 antihistamines. CGRP, calcitonin gene‐related peptide; mAb, monoclonal antibody.

During the study period, we investigated five cases of urticaria following treatment with anti‐CGRP mAbs. The patients' baseline characteristics are summarized in Table 1. Median age was 49 years (range 47–54 years), and all but one were female. Atopic comorbidities such as allergic rhinoconjunctivitis, asthma, and atopic dermatitis were present in four of five patients (Table 2). Three reactions were reported with erenumab, and two with fremanezumab (Table 2). One patient experienced angioedema in addition to wheals. One of the patients also received a short course of systemic corticosteroids to treat the initial reaction. Interestingly, three patients experienced urticaria after the first injection (Figure 1). Furthermore, the skin reactions typically occurred within 12–48 h, except in patient #3, who experienced urticaria and dizziness within 12 h of administration. In this patient, fremanezumab was continued prior to referral to the allergy clinic, and urticaria developed after all three injections, with increased severity and a shorter interval (3 h after the third injection).

**TABLE 1:** Baseline demographics and migraine‐related characteristics.

ID	Sex, Age	Initial CGRP‐mAb	Migraine form	Aura	Average monthly headache days at BL	Previous prophylactic migraine treatment	Medication overuse? (>10 d/month)
#1	m, 49	Erenumab	Chronic	No	30	Beta blocker, flunarizine, amitriptyline, duloxetine	No
#2	f, 47	Erenumab	Chronic	Yes	30	Beta blocker, amitriptyline	Yes
#3	f, 54	Fremanezumab	Chronic	Yes	16	Beta blocker, amitryptiline, lamotrigine, topiramate	Yes
#4	f, 47	Fremanezumab	Episodic	Yes	16	Beta blocker, lamotrigine	No
#5	f, 49	Erenumab	Chronic	Yes	30	Flunarizine, amitriptyline, lamotrigine	No

Abbreviations: BL, baseline; CGRP, calcitonin gene‐related peptide; f, female; ID, identifier; m, male; mAb, monoclonal antibody.

**TABLE 2:** Clinical reaction, management of re‐exposure, and outcome data.

ID	Initial CGRP mAb	N injection until reaction	Interval drug to reaction	Symptoms	Urticaria duration	Tryptase (BL)	Allergy testing with anti‐CGRP mAb	History of atopic diseases	History of urticaria or angio‐edema	Re‐exposure	Pre‐medication	Outcome
#1	Erenumab	1	20–40 h	Urticaria (W)	48 h	3.1 ng/mL	–	Asthma	Yes	Yes (erenumab)	Yes	Tolerated, still on mAb
#2	Erenumab	1	12 h	Urticaria (W)	24 h	6.9 ng/mL	–	RCA Asthma	Yes (CSU)	Yes (fremanezumab)	Yes	Tolerated, still on mAb
#3	Fremanezumab	1	>3–<12 h	Urticaria (W), dizziness	2 weeks	3.9 ng/mL	SPT/IDT/BAT negative	RCA AD	Yes	Yes (fremanezumab)	Yes	Tolerated, still on mAb
#4	Fremanezumab	3	24 h	Urticaria (L)	2 weeks	–	–	None	No	Yes (galcanezumab)	No	Local reaction, tolerated rimegepant
#5	Erenumab	6	24–48 h	Urticaria (W) angioedema	2 weeks	5.7 ng/mL	–	RCA AD	Yes	Yes (galcanezumab)	No	Angioedema stopped CGRP‐treatment

Abbreviations: AD, atopic dermatitis; BAT, basophil activation test; BL, baseline; CGRP, calcitonin gene‐related peptide; CSU, chronic spontaneous urticaria; h, hours; IDT, intradermal testing; L, localized, i.e. affecting just a single area of the body; RCA, allergic rhinoconjunctivitis; SPT, skin prick test; W, widespread urticaria.

Regarding symptom duration, urticaria resolved within 2 days in two patients and lasted about 2 weeks in the other three cases. Baseline mast cell tryptase levels were tested in four cases and were normal in all (Table 2). Other laboratory investigations were not performed systematically, consistent with current guidelines for management of acute urticaria.^14^ IgE‐mediated allergy was considered in patient #3, who experienced worsening of symptoms at shorter intervals after re‐exposure. She underwent skin prick and intradermal testing, as well as a basophil activation test with the culprit drug (fremanezumab), all of which were negative.

In all patients, re‐exposure to the same (*n* = 2) or an alternative anti‐CGRP mAb (*n* = 3) was performed (Figure 1). In three patients, including the two where the drug was not switched, re‐exposure was performed after premedication with an H1 antihistamine. Thus, premedication was not used systematically, but only after the occurrence of urticaria. As mentioned above, the premedication protocol was bilastine (20 mg orally) once daily starting 1 day before and continuing for 3 days after each anti‐CGRP mAb administration, as previously described for urticaria following mRNA COVID‐19 vaccination.^12^ None of the patients were given glucocorticoids as premedication. Patients were re‐exposed either to the same agent or switched to galcanezumab or fremanezumab (as detailed in Table 2). With premedication, CGRP‐mAb was tolerated in all three, regardless of whether the anti‐CGRP‐mAb was switched (Figure 1A–C). In patient #3, urticaria reoccurred upon stopping antihistamines (Figure 1C), requiring continuous antihistamine therapy, suggesting development of chronic urticaria. In the two patients who received another anti‐CGRP‐mAb without premedication, the same reaction occurred as with the culprit (Figure 1D,E). In patient #4, treatment was then switched to the oral anti‐CGRP rimegepant, which was well tolerated (Figure 1D). In contrast, patient #5 declined antihistamine premedication and wished to stop CGRP‐mAb therapy completely. Thus, anti‐CGRP therapy could be continued (follow‐up at least 3 months), despite initial urticaria in four of five patients, with two still on the initial drug (Figure 1A,C). Notably, all but one patient had previously experienced urticaria or angioedema triggered by other factors, including iron infusion, metamizole, seafood, or spontaneous urticaria (Table 2).

To further explore the frequency of urticaria to anti‐CGRP mAbs, we performed an inquiry of the pharmacovigilance data from the WHO database (VigiBase; ATC code N02CD [CGRP antagonists] and PT “urticaria”) on 17 October 2025, which did not reveal a statistically significant signal linking anti‐CGRP mAbs to urticaria: Information Component (IC) ranged from −0.7 to −2.8, and the lower 95% credibility limit (IC_25_) was <0 for all agents (threshold for a signal: IC_25_ > 0). Of note, 81% of individual urticaria reports during anti‐CGRP mAbs occurred in female, a proportion similar to that in our cohort.

## DISCUSSION

Urticaria is a common adverse event associated with monoclonal antibody treatments. According to the United States Food and Drug Administration's (FDA's) adverse events reporting system, about 6%–9% of reports related to CGRP‐mAb involved skin reactions,^10^, ^11^ mainly non‐serious injection‐site reactions. Urticaria was reported in 0.7%–1.6% with erenumab, 2.2%–2.7% with fremanezumab, 1.6%–1.8% with galcanezumab, and 0.6% with eptinezumab.^10^, ^11^ The data suggest that urticaria can occur across all CGRP‐mAbs. During the period of our study, erenumab and fremanezumab were the most frequently prescribed anti‐CGRP monoclonal antibodies at our center. The distribution of urticaria cases described here, therefore, reflects local prescribing patterns rather than comparative incidence. The available publications, however, provide no details on the clinical phenotype and potential risk factors. Moreover, data on the clinical approach to urticaria and the outcomes after reexposure are scarce. Our data show that, in typical cases with delayed urticaria, re‐exposure can be attempted with the same or an alternative anti‐CGRP mAb, with good tolerance when premedicated with H1 antihistamines.

IgE‐mediated reactions typically occur within minutes, to a maximum of 2 h, after exposure, following prior sensitization. Therefore, our observations about the occurrence of urticaria already after the first injection of an anti‐CGRP mAb in three of five patients combined with the long interval (12–24 h) until symptom onset suggest a non‐IgE‐mediated mechanism.^15^ Similar non‐IgE‐mediated reactions have been reported, especially with stabilizers such as polysorbate‐80, an ingredient in all four anti‐CGRP mAbs.^16^ The safe readministration of CGRP‐mAbs with an antihistamine premedication in three of three patients further supports a non‐IgE‐mediated mechanism. In non‐IgE‐mediated urticaria, treatment does not need to be stopped and may not require switching to an alternative CGRP‐mAb. Indeed, in our series, whether treatment was switched or not, reactions reoccurred unless an antihistamine was pre‐administered.

Our data do not exclude the possibility that urticaria may, in rare cases, also be triggered by IgE‐mediated hypersensitivity to CGRP‐mAbs. Therefore, patients should be evaluated by an allergist if reactions occur after previous tolerated administrations, when symptoms occur within a short time post‐administration (i.e., <6 h), when other signs of anaphylaxis, such as dyspnea or hypotension, are present, or when progressive symptoms occur following repeated injections.

Limitations of the study include the low number of patients, referral bias, the absence of a prospective study design with a control group, and a predefined diagnostic or management protocol. Findings may primarily apply to patients with a history of urticaria and may not be generalizable to all CGRP‐mAb‐treated patients. Therefore, our findings should be considered exploratory; and larger, prospective cohorts are needed to define the incidence and optimal management of anti‐CGRP‐associated urticaria.

As an alternative mechanism for urticaria, blocking CGRP may directly influence skin immunity. CGRP inhibits the function of inflammatory stimuli, T cells, and dendritic cells in the skin^17^, ^18^ and CGRP may also modulate directly mast cell activity and histamine release.^19^ Inhibition of CGRP may lower the threshold for histamine release and hives, particularly in individuals with a history of urticaria. In our case series, three patients had experienced previous episodes of urticaria triggered by drugs or food; however, the small sample size precludes a meaningful comparison of clinical phenotypes or outcomes between patients with and without such history. The exact relationship between delayed urticaria and CGRP‐mAbs, as well as potential risk factors like history of urticaria need to be investigated in larger patient cohorts. Notably, if urticaria occurs primarily in a specific patient subgroup, this might explain the lack of signal in the WHO pharmacovigilance database, as such events can be diluted when reports from the entire treated population are aggregated. Similarly, while anti‐CGRP therapies are being explored in inflammatory and dermatologic conditions, no conclusions regarding safety or efficacy in patients with underlying skin disease can be drawn from this case series. Our findings suggest that the occurrence of urticaria in such settings warrants careful monitoring. Finally, the potential mechanisms, such as the role of excipients like polysorbate‐80 or CGRP‐mediated immunomodulation, remain speculative. Systematic mechanistic investigations were beyond the scope of this retrospective case series and should be addressed in future studies.

In summary, our data indicate that urticaria occurring several hours after CGRP‐mAb injection is probably non‐IgE‐mediated, can successfully be prevented with premedication with H1 antihistamines and does not necessarily require a treatment switch. A history of urticaria or angioedema may present a risk factor for urticaria associated with CGRP‐mAb. Validation in larger cohorts will further delineate risk factors and support the development of evidence‐based management strategies.

## AUTHOR CONTRIBUTIONS

**Christoph T. Berger:** Conceptualization; methodology; investigation; data curation; visualization; writing – original draft; formal analysis. **Federico Burguet Villena:** Investigation; writing – review and editing; data curation. **Severin B. Vogt:** Investigation; writing – review and editing; methodology; formal analysis; resources. **Lukas Heydrich:** Writing – review and editing; resources; investigation. **Karin Hartmann:** Writing – review and editing; validation; supervision; resources. **Athina Papadopoulou:** Supervision; resources; formal analysis; writing – original draft; investigation; conceptualization; methodology.

## FUNDING INFORMATION

C.T.B., K.H., and A.P. received research funding from the Swiss National Science Foundation (SNSF; grant 310030_192440 to C.T.B.; grant 310030_207705 to K.H., and PZ00P3_216468 to A.P.). K.H. received additional research funding from the Swiss Cancer Research Foundation (grant KFS‐5979‐08‐2023), and the EU‐H2020‐MSCA‐COFUND EURIdoc program (No. 101034170). L.H. was supported by the Swiss National Science Foundation (Grants 33CM30‐124089), the UniBE ID research grant and the Cogito Foundation. F.B.V. was supported by a research grant (“Young talents in clinical research”) by the Swiss Academy of Medical Sciences and the G. & J. Bangerter Rhyner Foundation.

## CONFLICT OF INTEREST STATEMENT

**Christoph T. Berger** declares no conflict of interest. **Federico Burguet Villena** received travel support by TEVA. **Lukas Heydrich** received speaker fees/fees for advisory boards/consulting activities from Novartis and travel support from Teva. **Karin Hartmann** received speaker fees/fees for advisory boards/consulting activities from ALK, Allergopharma, Almirall, BioCryst, Blueprint, Cogent, Galderma, KalVista, Leo, Menarini, Novartis, Otsuka, Pfizer, Sanofi, Takeda and Thermo Fisher. **Athina Papadopoulou** received speaker fees/fees for advisory boards/consulting activities from Sanofi‐Genzyme, Eli Lilly, AbbVie, Lundbeck, Pfizer and TEVA and travel support from Bayer AG, Teva, AbbVie and Hoffmann‐La Roche. **Severin B. Vogt** has no conflicts to declare.

## Data Availability

Data will be made available upon reasonable request from qualified investigators. VigiBase, the WHO global database of reported potential side effects of medicinal products, developed and maintained by Uppsala Monitoring Centre is accessible under specific licensing terms. It is essential to emphasize that the data in VigiBase originate from various sources, and the probability of a suspected adverse effect being linked to a specific drug may vary across cases. The information presented in this article does not necessarily reflect the views or opinions of the UMC or the WHO. Access to VigiBase data was facilitated through the Vigilyze tool.
